# Supplementary material for: Mixing of meteoric and geothermal fluids supports hyperdiverse chemosynthetic hydrothermal communities
Source: Nat Commun. 2019 Feb 8;10:681. doi: 10.1038/s41467-019-08499-1 (PMC6368606; doi:10.1038/s41467-019-08499-1)
Supplement: Supplementary file 1 — Supplementary Information [file 41467_2019_8499_MOESM1_ESM.pdf]

Supplementary Figures and Tables

Mixing of meteoric and geothermal fluids supports hyperdiverse chemosynthetic hydrothermal communities

Colman, *et al.*

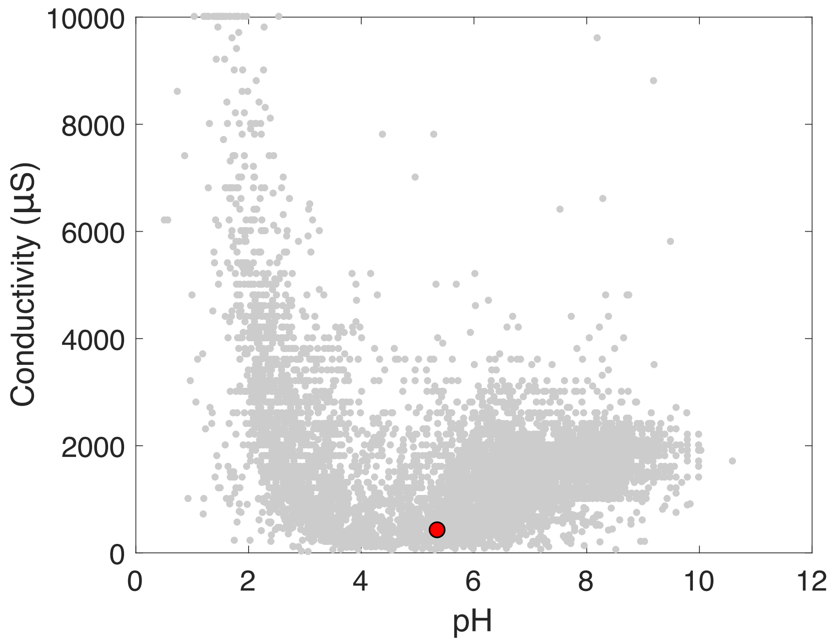

Supplementary Figure 1. Conductivity and pH values of SJ3 in context of those for YNP thermal springs. Conductivity and pH measurements for hot springs reported in the YNP thermal inventory (n=6850) available in the Research Coordination Network database (http://www.rcn.montana.edu) are plotted as grey circles. 'SJ3' is plotted in red.

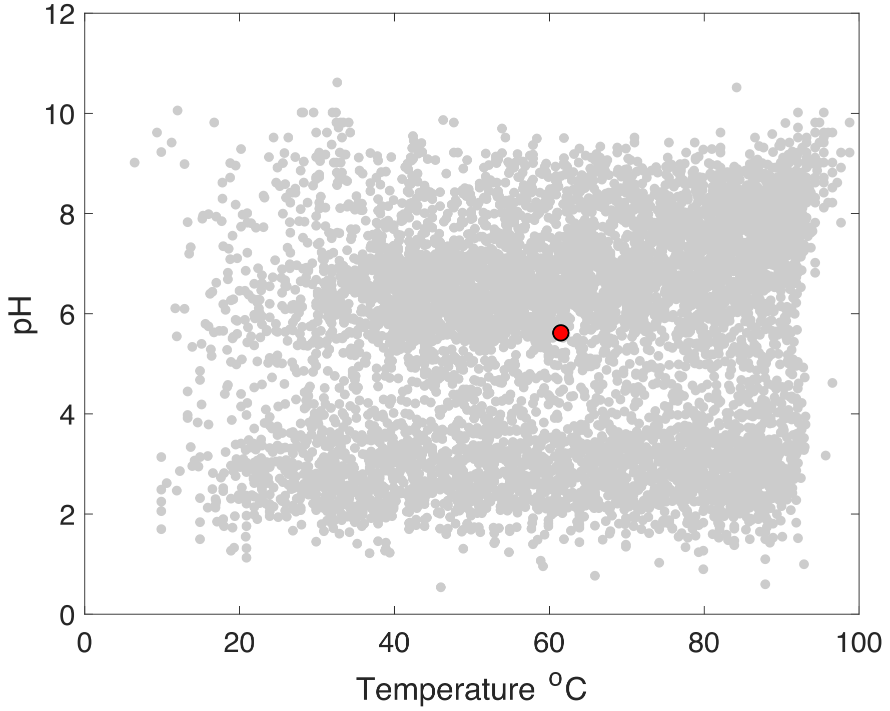

Supplementary Figure 2. Temperature and pH of spring waters from SJ3 in context of those for other YNP thermal springs. Temperature and pH measurements for hot springs reported in the YNP thermal inventory (n=7693) available in the Research Coordination Network database (http://www.rcn.montana.edu) are plotted as grey circles. 'SJ3' is plotted in red.

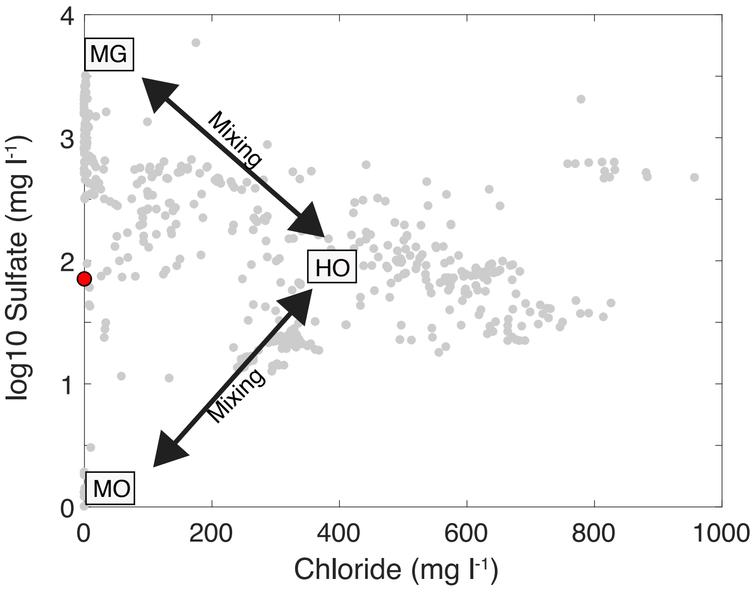

Supplementary Figure 3. Sulfate and chloride values of SJ3 in context of those for other YNP springs. Sulfate and chloride data from hot springs in YNP as reported in Ball *et al.*, 2006 and McCleskey *et al.,* 2014 are plotted as grey circles (n=488). 'SJ3' is plotted in red. Plotting of the data follows Nordstrom *et al.* 2009[23] wherein HO=hydrothermal only waters theorized to be sourced from a deep hydrothermal reservoir, MO=dilute meteoric or precipitation derived waters, and MG=acid-sulfate waters that represent meteoric waters influenced by vapor-phase gas inputs. Springs that are right-shifted from the HO group represent deep waters that are interpreted to have also undergone subsurface boiling and evaporation resulting in a higher concentration of chloride (hydrothermal fluids with subsurface boiling, 'HB', in Nordstrom *et al.* 2009[23]). Source data are provided within the source data file accompanying this manuscript.

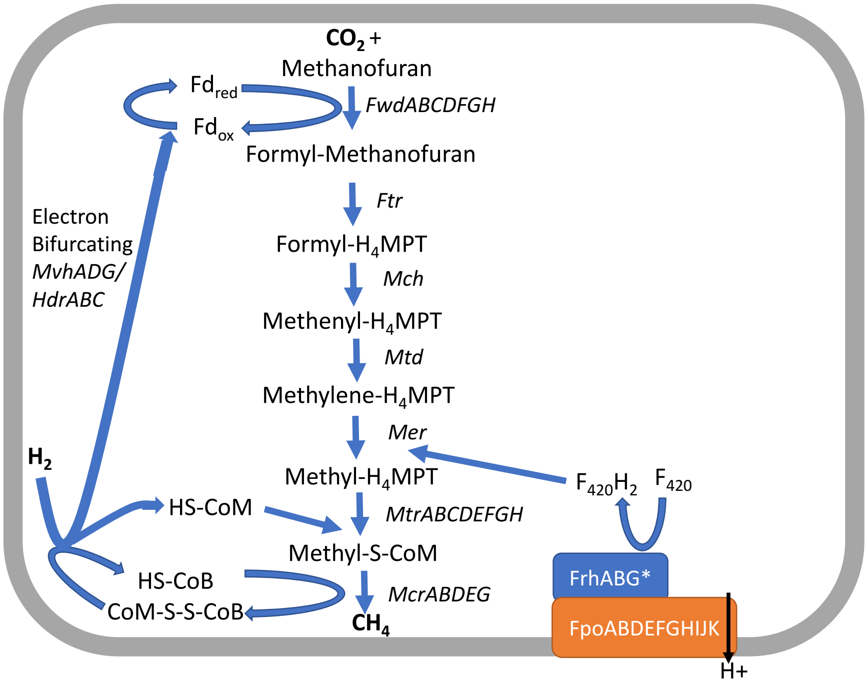

Supplementary Figure 4. Schematic of inferred methanogenesis pathway based on the Archaeoglobales Bin34 MAG. Substrates and products of methanogenesis are indicated in bold, while enzymes involved in each step are indicated in italics. Fwd, formylmethanofuran dehydrogenase; Ftr, formylmethanofuran tetrahydromethanopterin formyltransferase; Mch, methenyltetrahydromethanopterin cyclohydrolase; Mtd, methylenetetrahydromethanopterin dehydrogenase, Mer, methylenetetrahydromethanopterin reductase; Mtr, methyl-H_4MPT:HS-CoM methyltransferase; Mcr, methyl coenzyme-M reductase; Mvh, F_{420} non-reducing [NiFe]-hydrogenase (group 3c); Hdr, heterodisulfide reductase; Fpo, $F_{420}H_2$:phenazine oxidoreductase ; $Fd_{red/ox}$, ferredoxin (reduced/oxidized); CoM, coenzyme-M; CoB, coenzyme-B; Frh, F_{420} reducing hydrogenase. FrhABG* indicates hypothetical position as FpoD lacks the characteristic N- and C-terminus cysteinyl residues present in the FpoD of methanogens that either reduce ferredoxin or $F_{420}H_2$ with Fpo[58]. In contrast, the F_{420} reductase subunit (FrhB) of the F_{420} reducing hydrogenase was co-localized with the Fpo operon in the genome, but not with the F_{420} reducing hydrogenase large (catalytic) and small subunits. Consequently, we speculate that FrhB

reduces F_{420} and interacts with the membrane bound Fpo complex, replacing the catalytic role of FpoD in other methanogens (e.g., *Methanosarcina barkeri*).

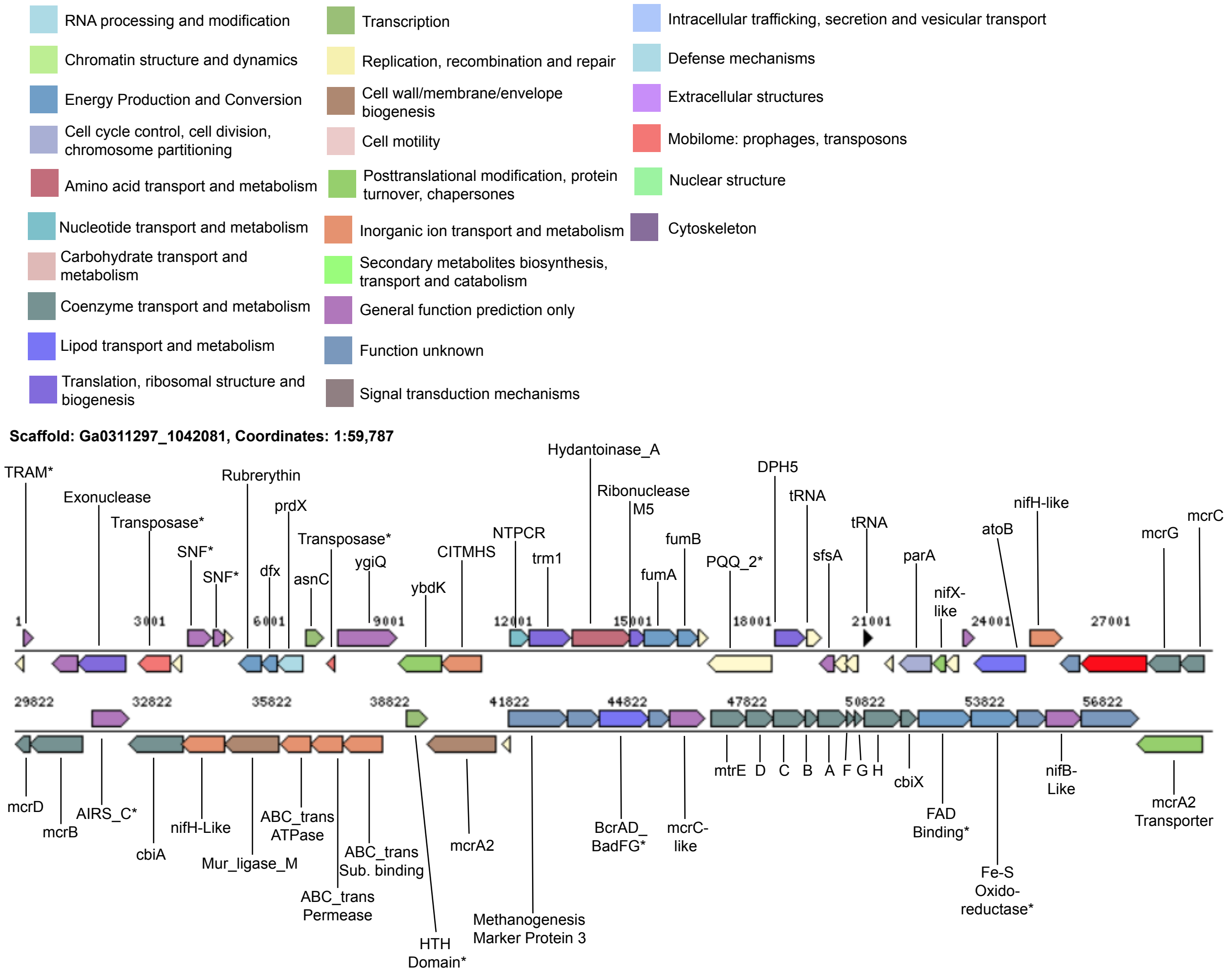

Supplementary Figure 5. Mcr protein coding gene neighborhood of the Archaeoglobales-affiliated SJ3.Bin34. The McrA homolog is indicated in red within the scaffold GA0311297_1042081, while other genes are colored according to their general Clusters of Orthologous Groups (COG) category, as denoted above the gene neighborhood visualization. The gene neighborhood was drawn with visualization tools in the IMG web server. Gene names are provided, or otherwise the protein domain present within the inferred gene is indicated by an asterisk. Inferred hypothetical proteins without assigned functions or domains are unlabeled.

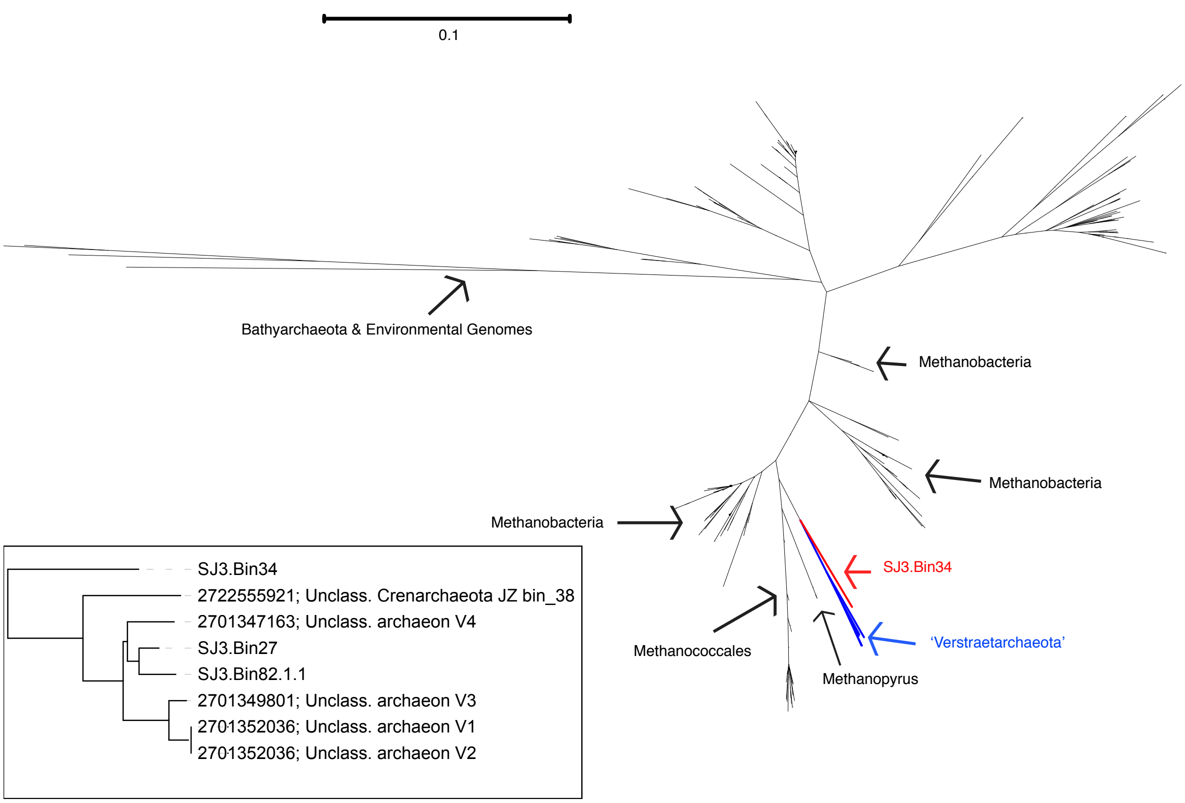

Supplementary Figure 6. Phylogenetic placement of McrA from three SJ3 archaeal genome bins. The McrA phylogeny was produced using Maximum-Likelihood approaches on aligned McrA proteins. The lineage highlighted in red depict the placement of the McrA from SJ3.Bin34, while the lineages in blue represent the *Ca.* 'Verstraetarchaeota' McrA, including those from SJ3.Bin27 and SJ3.Bin82.1.1. The inset image depicts a zoomed in view of the SJ3.Bin34/ *Ca.* 'Verstraetarchaeota' McrA clade, with IMG gene numbers given for each reference McrA.

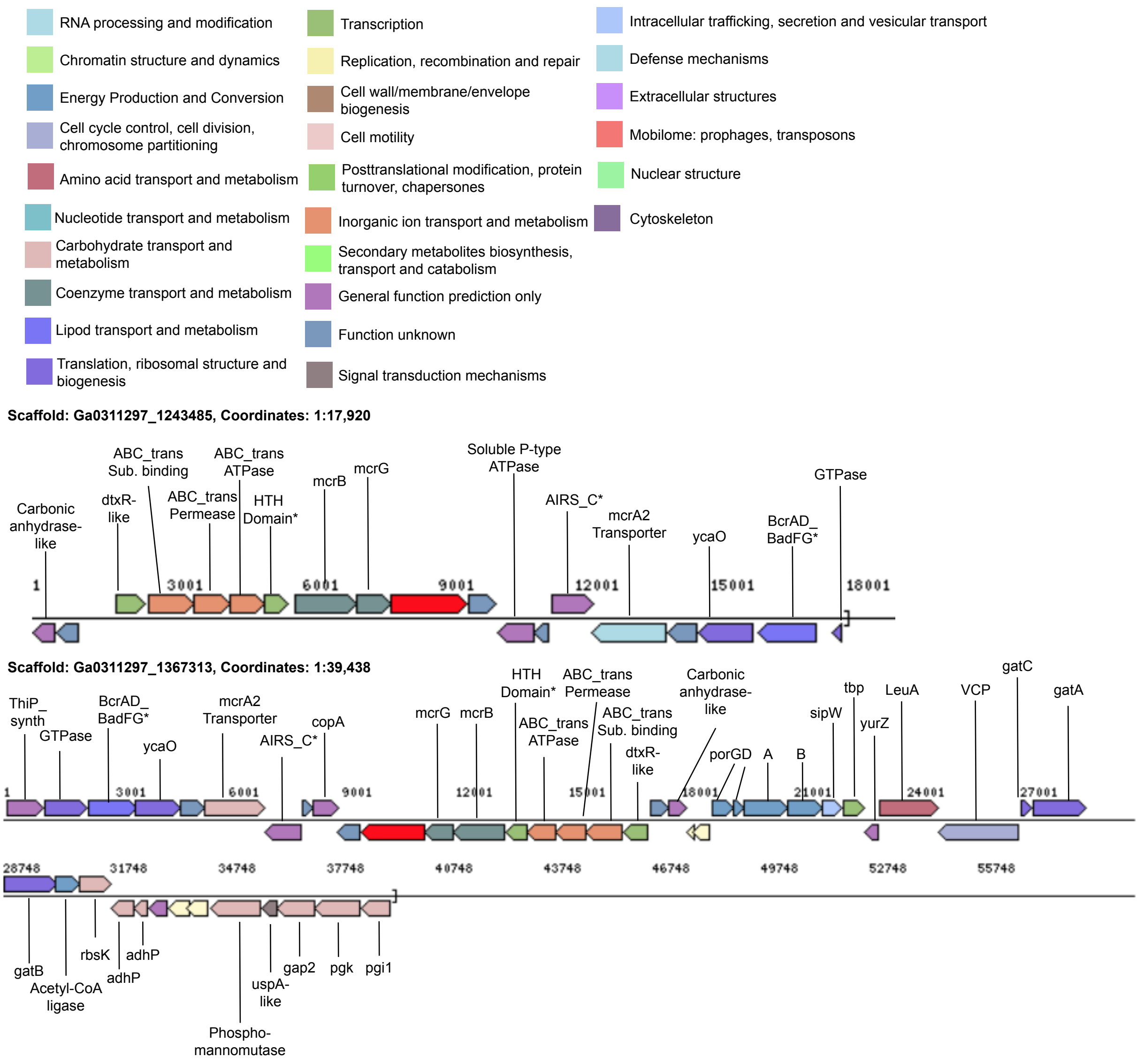

Supplementary Figure 7. Mcr protein coding gene neighborhood of the *Ca.* 'Verstraetarchaeota'-affiliated SJ3.Bin27 (top) and SJ3.Bin82.1.1 (bottom). The McrA homolog is indicated in red within scaffold GA0311297_123485 (SJ3.Bin27) and GA0311297_1367313 (SJ3.Bin82.1.1), while other genes are colored according to their general Clusters of Orthologous Groups (COG) category, as denoted above the gene neighborhood visualization. The gene neighborhood was drawn with visualization tools in the IMG web server. Gene names are provided, or otherwise the protein domain present within the inferred gene is

indicated by an asterisk. Inferred hypothetical proteins without assigned functions or domains are unlabeled.

Supplementary Table 1. SJ3 Metagenome Assembly Information

Total Reads (x 10^6)	290.69
Total Sequencing Effort (Gbp)	60.75
Total assembled sequence >500 bp (Mbp)	278.41
Total assembled sequence >10,000 bp (Mbp)	86.87
N50 (bp)	3,907.00
Proteins (Binned)	298,127.00
rRNA genes (>300 bp length)	259.00
Contigs (>500 bp)	140,367.00
Contigs > 1000 bp	58,965.00
Contigs > 25000 bp	931.00
Longest Contig (bp)	438,391.00
