# Supplementary material for: Mixing of meteoric and geothermal fluids supports hyperdiverse chemosynthetic hydrothermal communities
Source: Nat Commun. 2019 Feb 8;10:681. doi: 10.1038/s41467-019-08499-1 (PMC6368606; doi:10.1038/s41467-019-08499-1)
Supplement: Supplementary file 3 — Description of Additional Supplementary Files [file 41467_2019_8499_MOESM3_ESM.pdf]

Description of Additional Supplementary Files

File Name: Supplementary Data File 1

Description: Full archaeal phylogeny with taxonomic annotations.

File Name: Supplementary Data File 2

Description: Full bacterial phylogeny with taxonomic annotations.

File Name: Supplementary Data File 3

Description: Relative enrichment values for Kegg Orthology (KO) protein families among SJ3 and 14 other characterized chemosynthetic YNP spring metagenomes. Values of -1 indicate absence, while higher values indicate higher relative enrichment for each KO within a metagenome. Relative enrichment calculations were conducted as described in the materials and methods.

File Name: Supplementary Data File 4

Description: Coverage and G+C content coverage profiles for archaeal genome bins belonging to the same divisions that were not shown in Figure 7.

File Name: Supplementary Data File 5

Description: Coverage and G+C content coverage profiles for bacterial genome bins belonging to the same divisions.

File Name: Supplementary Data File 6

Description: Additional contig, protein coding gene, and KEGG mapping information for the SJ3 MAGs associated with the Archaeoglobales.

File Name: Supplementary Data File 7

Description: Additional contig, protein coding gene, and KEGG mapping information for the SJ3 MAGs associated with the Ca. 'Verstraetarchaeota'.

File Name: Supplementary Data File 8

Description: Additional contig, protein coding gene, and KEGG mapping information for the SJ3 MAGs associated with the Thaumarchaeota.

File Name: Supplementary Data File 9

Description: Genome bin information for the MAGs generated in this study that were estimated to be >50% complete.

File Name: Supplementary Data File 10

Description: Metadata, geochemical information, and citations for chemosynthetic community metagenomes from YNP that were used for comparison against SJ3.
