# Supplementary figures and images for: Mixing of meteoric and geothermal fluids supports hyperdiverse chemosynthetic hydrothermal communities
Source: Nat Commun. 2019 Feb 8;10:681. doi: 10.1038/s41467-019-08499-1 (PMC6368606; doi:10.1038/s41467-019-08499-1)

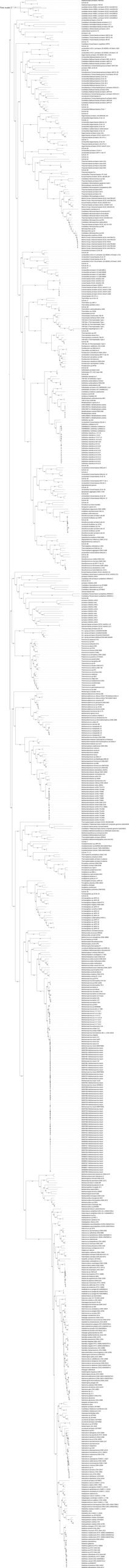

Supplement: Supplementary file 4 — Supplementary Data 1 [file 41467_2019_8499_MOESM4_ESM.pdf]

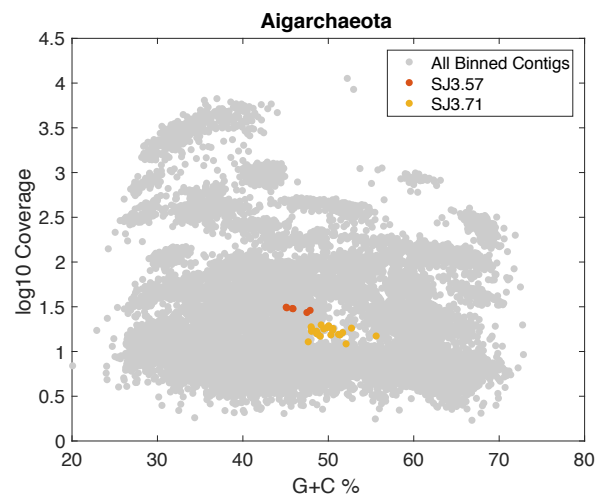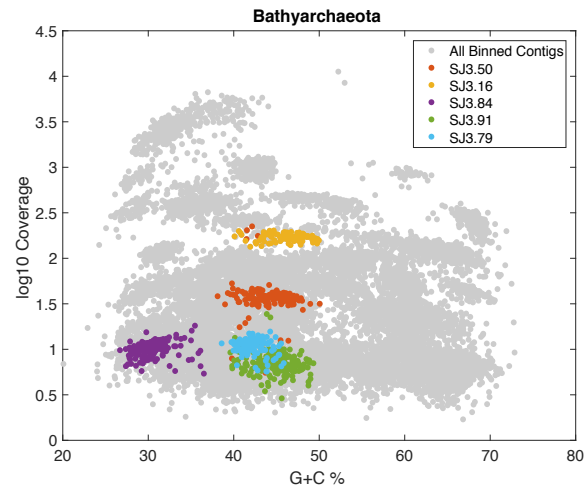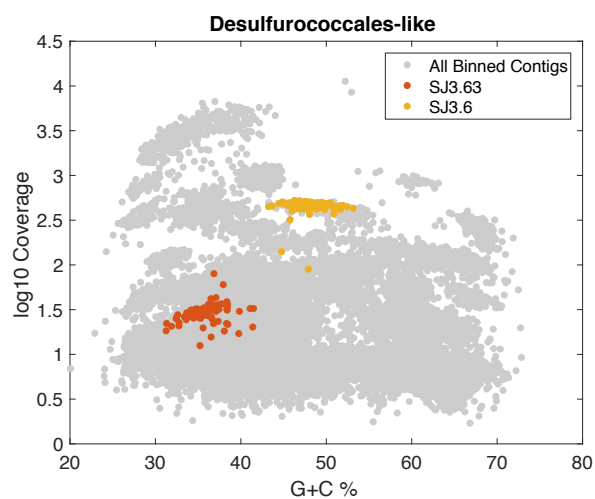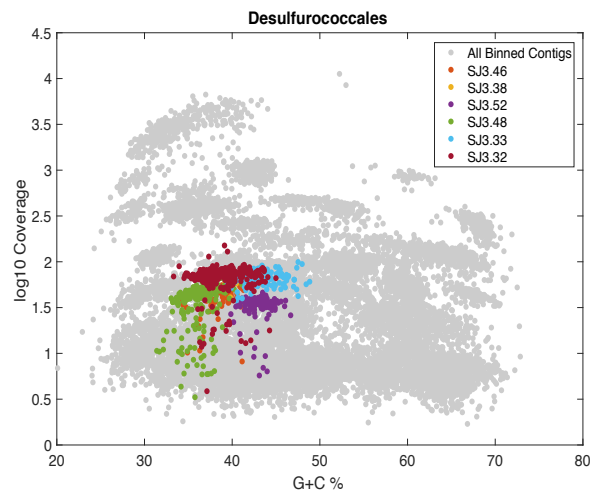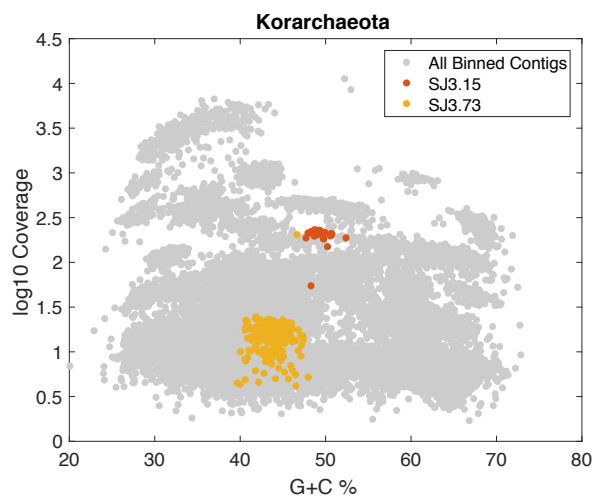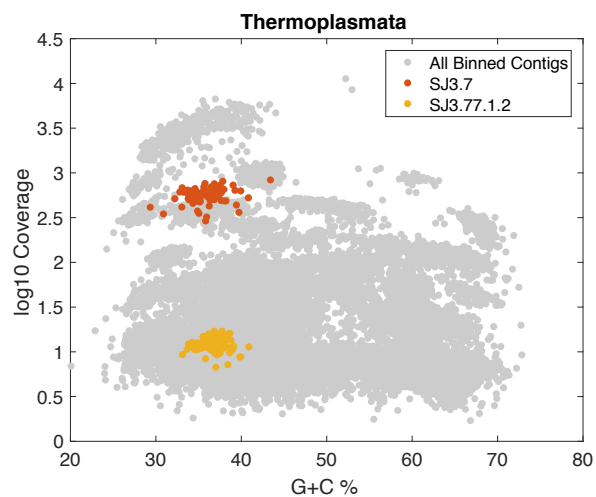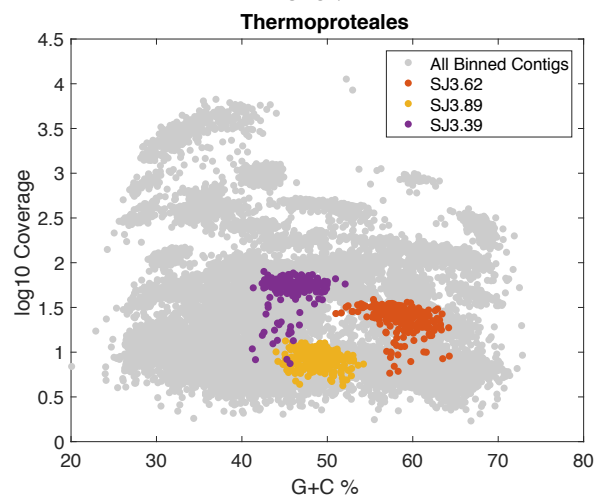

Supplement: Supplementary file 7 — Supplementary Data 4 [file 41467_2019_8499_MOESM7_ESM.pdf]

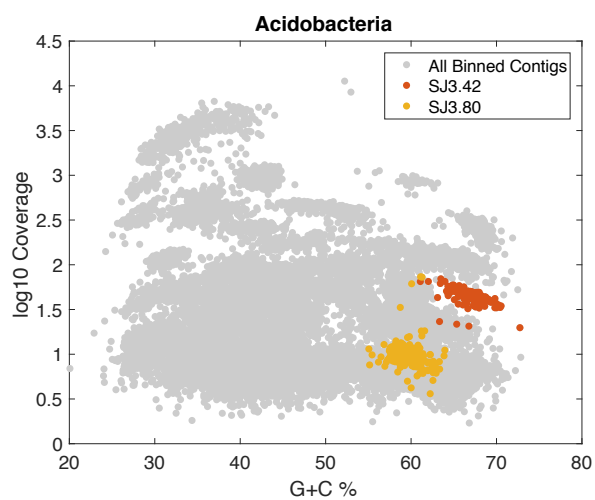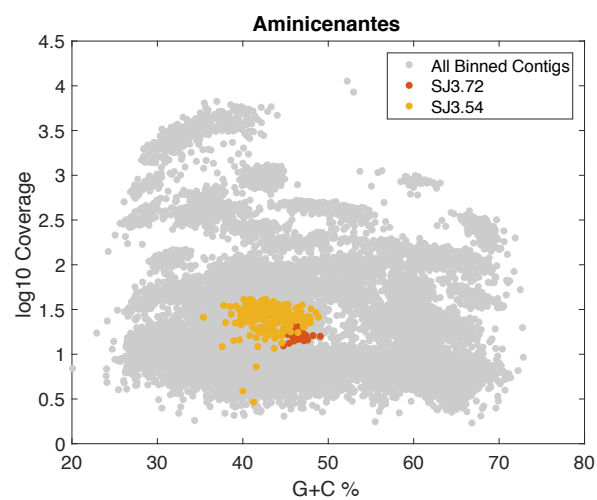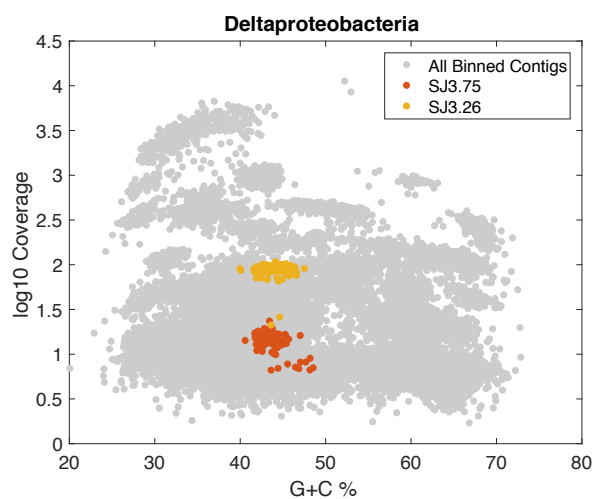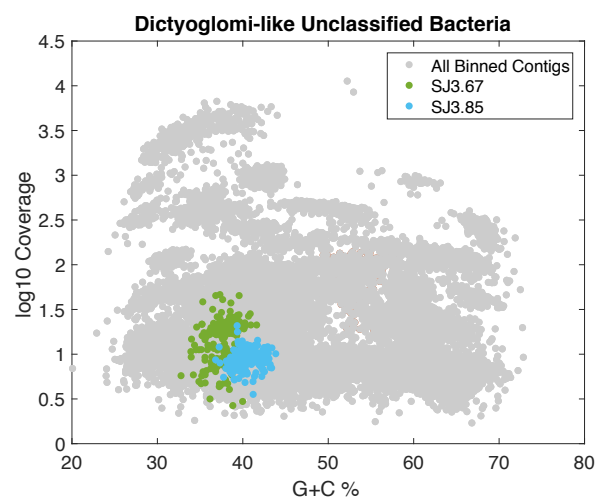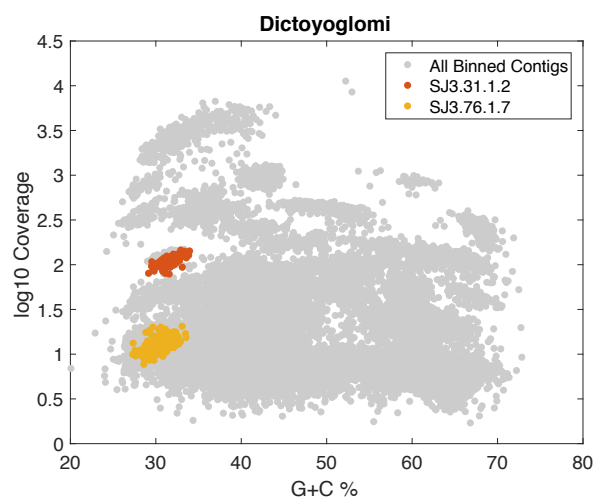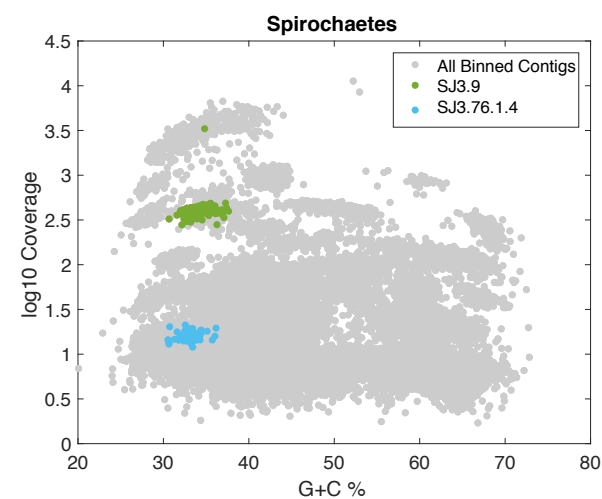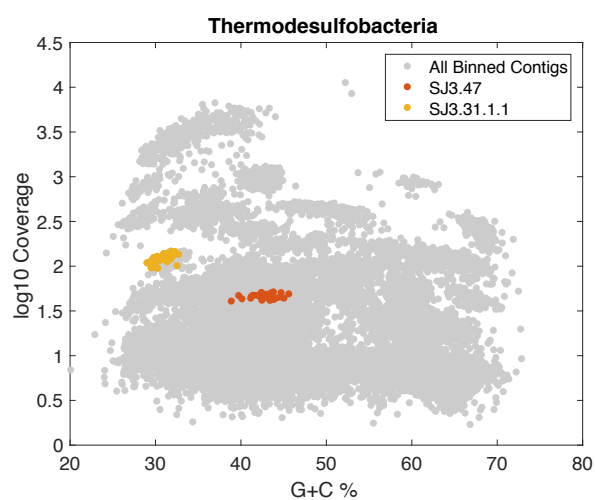

Supplement: Supplementary file 8 — Supplementary Data 5 [file 41467_2019_8499_MOESM8_ESM.pdf]
