# Supplementary material for: Mixing of meteoric and geothermal fluids supports hyperdiverse chemosynthetic hydrothermal communities
Source: Nat Commun. 2019 Feb 8;10:681. doi: 10.1038/s41467-019-08499-1 (PMC6368606; doi:10.1038/s41467-019-08499-1)
Supplement: Supplementary file 5 — Supplementary Data 2 [file 41467_2019_8499_MOESM5_ESM.pdf]

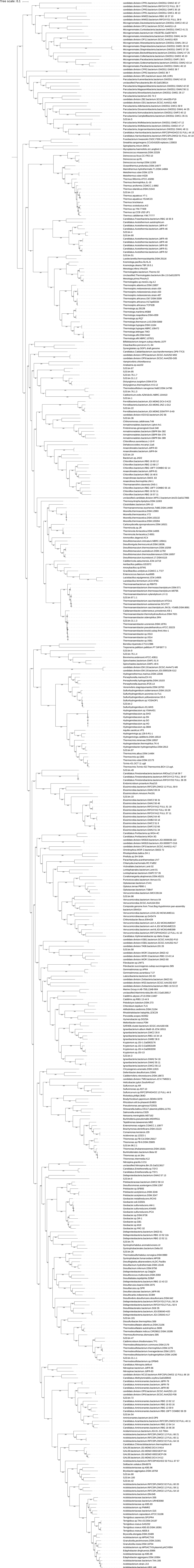
Tree scale: 0.1
