# Supplementary material for: Mixing of meteoric and geothermal fluids supports hyperdiverse chemosynthetic hydrothermal communities
Source: Nat Commun. 2019 Feb 8;10:681. doi: 10.1038/s41467-019-08499-1 (PMC6368606; doi:10.1038/s41467-019-08499-1)
Supplement: Supplementary file 14 — Reporting Summary [file 41467_2019_8499_MOESM14_ESM.pdf]

nature research

Corresponding author(s): Eric Boyd

Reporting Summary

Nature Research wishes to improve the reproducibility of the work that we publish. This form provides structure for consistency and transparency in reporting. For further information on Nature Research policies, see Authors & Referees and the Editorial Policy Checklist.

Statistical parameters

When statistical analyses are reported, confirm that the following items are present in the relevant location (e.g. figure legend, table legend, main text, or Methods section).

n/a	Confirmed	
☐	☒	The exact sample size (*n*) for each experimental group/condition, given as a discrete number and unit of measurement
☐	☒	An indication of whether measurements were taken from distinct samples or whether the same sample was measured repeatedly
☐	☒	The statistical test(s) used AND whether they are one- or two-sided<br>*Only common tests should be described solely by name; describe more complex techniques in the Methods section.*
☒	☐	A description of all covariates tested
☐	☒	A description of any assumptions or corrections, such as tests of normality and adjustment for multiple comparisons
☐	☒	A full description of the statistics including central tendency (e.g. means) or other basic estimates (e.g. regression coefficient) AND variation (e.g. standard deviation) or associated estimates of uncertainty (e.g. confidence intervals)
☒	☐	For null hypothesis testing, the test statistic (e.g. *F*, *t*, *r*) with confidence intervals, effect sizes, degrees of freedom and *P* value noted<br>*Give P values as exact values whenever suitable.*
☒	☐	For Bayesian analysis, information on the choice of priors and Markov chain Monte Carlo settings
☒	☐	For hierarchical and complex designs, identification of the appropriate level for tests and full reporting of outcomes
☒	☐	Estimates of effect sizes (e.g. Cohen's *d*, Pearson's *r*), indicating how they were calculated
☒	☐	Clearly defined error bars<br>*State explicitly what error bars represent (e.g. SD, SE, CI)*

Our web collection on statistics for biologists may be useful.

Software and code

Policy information about availability of computer code

Data collection

The metagenomic data collected for this manuscript were generated using the manufacturer pipelines on the HiSeq Rapid platforms (SJ3 spring).

Data analysis

As detailed in the methods section, the software program Trimmomatic (v.0.35) was used to clean raw metagenomic sequence reads, while MEGAHIT (v.1.1.1) and metaquast (v.3.2) were used to assemble the clean reads into contigs, and assess the quality of the assemblies, respectively. Bowtie2 was used to map raw reads to assembled contigs to generate coverage depth profiles. Assembled contigs were binned to draft metagenome assembled genome (MAG) bins using the Metabat (v.0.26.3) software package, while the CheckM software suite (v.1.0.5) was used to determine MAG quality and completeness. CONCOCT v.0.4.1 was also used to bin contigs into MAGs and these data were compared against those produced by the Metabat program. MAG protein coding genes were annotated with the annotation software program PROKKA (v.1.11). Phylogenetic analyses were conducted using the RAxML (v.8.2.9).

In some cases, the completeness of genome bins that were estimated to be abundant (> 1.0% estimated relative abundance) exhibited low completeness, as is common in deep metagenomic sequencing of environmental genomic DNA. Improvement in the quality of these bins was attempted by recruiting contigs to genomes that were publicly available or were available from other in-house metagenomes and shared high marker protein identity to the low-completeness bins. Contig recruitment was conducted with the MG Wrapser tool (https://github.com/dunfieldlab/mg_wrapser), followed by extraction of quality-filtered reads mapped to the contigs using the Multi-metagenome package (v.1.0). Extracted reads were then reassembled using the Spades v.3.10.0 assembler, which produced higher quality assemblies than MEGAHIT for individual populations. Assessment of assembly quality and population homogeneity was conducted with MetaQUAST and CheckM, and final genome bins were curated as described above using RefineM (v.0.0.22).

For manuscripts utilizing custom algorithms or software that are central to the research but not yet described in published literature, software must be made available to editors/reviewers upon request. We strongly encourage code deposition in a community repository (e.g. GitHub). See the Nature Research guidelines for submitting code & software for further information.

Data

Policy information about availability of data

All manuscripts must include a data availability statement. This statement should provide the following information, where applicable:

- Accession codes, unique identifiers, or web links for publicly available datasets
- A list of figures that have associated raw data
- A description of any restrictions on data availability

Assembled contigs have been uploaded to the Integrated Microbial Genomes (IMG) database under the genome ID 3300029625 (public release pending final acceptance).

Field-specific reporting

Please select the best fit for your research. If you are not sure, read the appropriate sections before making your selection.

☐ Life sciences ☐ Behavioural & social sciences ☒ Ecological, evolutionary & environmental sciences

For a reference copy of the document with all sections, see nature.com/authors/policies/ReportingSummary-flat.pdf

Ecological, evolutionary & environmental sciences study design

All studies must disclose on these points even when the disclosure is negative.

Study description	The study was designed to assess the role of end member fluid mixing on the taxonomic and functional diversity present within SJ3 spring within Yellowstone National Park. To contextualize the levels of diversity present in the spring, comparisons were made to all publicly available metagenomes that were considered to be chemosynthetic based on published or other available data.
Research sample	All original DNA sequence data within the manuscript are derived from sediment samples taken from the SJ3 spring. Sediments were collected sterily and frozen on dry ice immediately, before storing in a -80 freezer upon return to the laboratory.
Sampling strategy	The sample for metagenomic analyses was chosen based on the geochemical attributes that were associated with previous geochemical analysis of the spring (i.e., high levels of hydrogen and other gases). All publicly available chemosynthetic community metagenomes from YNP were used for comparison. Metagenomes were excluded that did not appear to be natural samples (e.g., they were engineered or enrichment samples), or appeared to represent duplicate metagenomes to those within the dataset.
Data collection	Total genomic DNA from SJ3 sediments were shotgun sequenced at the Genomics Core Facility at the University of Wisconsin-Madison using the paired-end (2 x 250 bp) Illumina HiSeq platform. All publicly available chemosynthetic community metagenomes from YNP were used for comparison from the IMG web server.
Timing and spatial scale	SJ3 sediments were collected in July 2014, and relevant geochemical data are provided in the manuscript, or in another paper that is currently under review (Lindsey et al.). SJ3 sediments have not been re-sampled due to the difficulty in accessing the Smokejumper geyser basin (~20 mile round trip hike at high elevation that is not snow-covered for only a short period during the summer).
Data exclusions	Data were not excluded from the analyses described here.
Reproducibility	To assess whether the observations made at SJ3 were consistent across other geyser basins and hot springs, a broader comparison to all publicly available chemosynthetic spring communities of YNP was conducted.
Randomization	Randomization strategies were not relevant to the experimental design, because the data derives from one sample set.

Blinding	Blinding was not relevant to this study.

Did the study involve field work? ☒ Yes ☐ No

Field work, collection and transport

Field conditions	SJ3 sediments were collected in July 2014, and relevant geochemical data are provided in the manuscript, or in a paper that is currently under review at ISMEJ (Lindsey et al.).
Location	Sediments were sampled from SJ3 spring that is located within the Smokejumper Geyser Basin (N 44°24'57.42"; W -110° 57'20.76") of Yellowstone National Park in July 2014.
Access and import/export	Sediment samples for SJ3 spring were collected under a sampling permit for Yellowstone National Park issued to Eric S. Boyd. DNA is archived at the Boyd laboratory at Montana State University.
Disturbance	Disturbance to the system was not introduced by this study, as only small (<5 g) samples of sediments were taken for DNA analyses.

Reporting for specific materials, systems and methods

Materials & experimental systems

n/a	Involved in the study	
☐	☒	Unique biological materials
☒	☐	Antibodies
☒	☐	Eukaryotic cell lines
☒	☐	Palaeontology
☒	☐	Animals and other organisms
☒	☐	Human research participants

Methods

n/a	Involved in the study	
☒	☐	ChIP-seq
☒	☐	Flow cytometry
☒	☐	MRI-based neuroimaging

Unique biological materials

Policy information about availability of materials

Obtaining unique materials	Sediment samples for SJ3 spring were collected under a sampling permit for Yellowstone National Park issued to Eric S. Boyd.
